# The *SOC1*-like gene *BoMADS50* is associated with the flowering of *Bambusa oldhamii*

**DOI:** 10.1038/s41438-021-00557-4

**Published:** 2021-06-01

**Authors:** Dan Hou, Ling Li, Tengfei Ma, Jialong Pei, Zhongyu Zhao, Mengzhu Lu, Aimin Wu, Xinchun Lin

**Affiliations:** 1 State Key Laboratory of Subtropical Silviculture, Zhejiang A & F University, Lin’an, 311300 Hangzhou, China; 2 State Key Laboratory for Conservation and Utilization of Subtropical Agro-bioresources, South China Agricultural University, 510642 Guangzhou, China; 3 Guangdong Key Laboratory for Innovative Development and Utilization of Forest Plant Germplasm, College of Forestry and Landscape Architecture, South China Agricultural University, 510642 Guangzhou, China

**Keywords:** Plant sciences, Molecular biology

## Abstract

Bamboo is known for its edible shoots and beautiful texture and has considerable economic and ornamental value. Unique among traditional flowering plants, many bamboo plants undergo extensive synchronized flowering followed by large-scale death, seriously affecting the productivity and application of bamboo forests. To date, the molecular mechanism of bamboo flowering characteristics has remained unknown. In this study, a *SUPPRESSOR OF OVEREXPRESSION OF CONSTANS1* (*SOC1*)-like gene, *BoMADS50*, was identified from *Bambusa oldhamii*. *BoMADS50* was highly expressed in mature leaves and the floral primordium formation period during *B. oldhamii* flowering and overexpression of *BoMADS50* caused early flowering in transgenic rice. Moreover, BoMADS50 could interact with APETALA1/FRUITFULL (AP1/FUL)-like proteins (BoMADS14-1/2, BoMADS15-1/2) in vivo, and the expression of *BoMADS50* was significantly promoted by BoMADS14-1, further indicating a synergistic effect between BoMADS50 and BoAP1/FUL-like proteins in regulating *B. oldhamii* flowering. We also identified four additional transcripts of *BoMADS50* (*BoMADS50-1/2/3/4*) with different nucleotide variations. Although the protein-CDS were polymorphic, they had flowering activation functions similar to those of *BoMADS50*. Yeast one-hybrid and transient expression assays subsequently showed that both *BoMADS50* and *BoMADS50-1* bind to the promoter fragment of itself and the *SHORT VEGETATIVE PHASE* (*SVP*)-like gene *BoSVP*, but only *BoMADS50-1* can positively induce their transcription. Therefore, nucleotide variations likely endow *BoMADS50-1* with strong regulatory activity. Thus, *BoMADS50* and *BoMADS50-1/2/3/4* are probably important positive flowering regulators in *B. oldhamii*. Moreover, the functional conservatism and specificity of *BoMADS50* and *BoMADS50-1* might be related to the synchronized and sporadic flowering characteristics of *B. oldhamii*.

Woody bamboos are tree-like woody grasses and serve as an economically important resource worldwide because they produce timber, fiber, food, and other products^1^. Among these, *Bambusa oldhamii* is famous for its delicious and nutritious shoots. This plant is highly adaptive and shows strong regeneration, and the shooting period can last for 5 months per year. The annual output of *B. oldhamii* shoots reaches up to 10,000 tons, which have significant economic value. Bamboo has a unique flowering characteristic and unpredictable vegetative period^2^. These plants generally die after blooming, resulting in reduced production or even the death of shoots, which seriously affects the productivity, development, and ecosystem of bamboo forests. Consequently, increasing attention has been given to bamboo flowering for its scientific importance and crucial role in some human communities.

The flowering cycles of different bamboo species vary. For example, the flowering time of *Phyllostachys* is 13–120 years, while that of *Bambusa* is 30–150+ years^3–5^. Interestingly, although the flowering of bamboo has a strong periodicity, some bamboo species have flowering habits that are extensively synchronized and sporadic^4,6^. For example, the majority of *B. oldhamii* do not flower until ~30–60 years of age^7^, but even in a bamboo population formed by the same clone, some clusters or branches frequently blossom sporadically^4,8^. Many hypotheses about the bamboo flowering cycle have emerged^3,4,9^. In recent years, the completion of genome sequencing of *Phyllostachys edulis*^10,11^, transcriptome sequencing of different bamboo species^12^, and functional analysis of bamboo flowering genes^7,13^ have provided a crucial basis for bamboo flowering studies. However, until now, “the molecular aspects of bamboo flowering remain at a nascent stage”^14^, and why many bamboo species bloom periodically and sporadically is still unclear.

MADS transcription factors are best known for their involvement in determining floral development^15–18^. In addition to their functional roles as floral organ identity genes, *MADS* genes also participate in floral induction^19–21^. In *Arabidopsis thaliana*, *SUPPRESSOR OF OVEREXPRESSION OF CONSTANS1*/*AGAMOUS-LIKE 20* (*SOC1*/*AGL20*) belongs to the MIKC (MADS DNA-binding domain, intervening domain, keratin-like domain, and C-terminal domain)-type MADS-box gene family^22^, constitutes a major hub in a complex regulatory network underlying floral timing and plays a central role in *A. thaliana* reproduction by integrating signals of the photoperiod, autonomous, vernalization, age, and gibberellin pathways^23,24^. The *SOC1-*like gene also exists as a flowering activator in many plant species, including *Zea mays*^25^, *Medicago truncatula*^26^, and *Eriobotrya japonica*^27^. In rice, *OsMADS50*, a gene highly homologous to *A. thaliana SOC1*, functions as a long-day (LD)-specific flowering promoter mainly through the *Oryza sativa* LEC2 and FUSCA3 Like 1-Early heading date 1-Heading date 3a/RICE FLOWERING LOCUS T1 (OsLFL1-Ehd1-Hd3a/RFT1) pathways^28,29^. This gene may be regulated by the *FLO/LFY homolog of rice* (*RFL*) and function parallel to other rice flowering time genes, such as *Heading date 1* (*Hd1*) and *GIGANTEA* (*GI*), to eventually activate the expression of *the FLOWERING LOCUS T* (*FT*)-like gene^30^. Therefore, “a proper integration of inductive floral cues by *SOC1-*like genes is an important step in regulating plants floral induction”^26^.

In woody bamboos, the *SOC1-*like gene is considered one of the potential factors influencing vegetative growth and floral timing^2^. This finding is mainly because the protein structures of woody bamboo SOC1-like proteins are clearly different from those of herbaceous plants (with annual flowering behavior, such as rice and other typical grasses), which may lead to the loss/malfunction of *SOC1-*like genes and even strong variations in flowering time^2^. Additionally, the overexpression of the *SOC1*-like genes *PvSOC1* and *PvMADS56* from *P. violascens* can significantly promote flowering in transgenic *A. thaliana* or rice, indicating their essential roles in bamboo flowering determination^31,32^. Therefore, understanding the function of the *SOC1-*like gene is necessary for the elucidation of the flowering mechanism in woody bamboo.

In the current study, we reported the nuclear-localized SOC1-like protein BoMADS50 from *B. oldhamii*. BoMADS50 is likely involved in bamboo flowering induction by acting in coordination with BoAP1/FUL proteins. Moreover, the other four *BoMADS50* transcripts (*BoMADS50-1/2/3/4*) with different nucleotide variations might be functionally conserved as flowering activators. However, unlike *BoMADS50*, *BoMADS50-1* showed strong regulatory activity toward target genes. In brief, our data define a putative regulatory pathway of *BoMADS50* and provide a new scientific hypothesis for massive bamboo and sporadic flowering regulation.

## Results

### Cloning and identification of *BoMADS50*

**Fig. 1:**
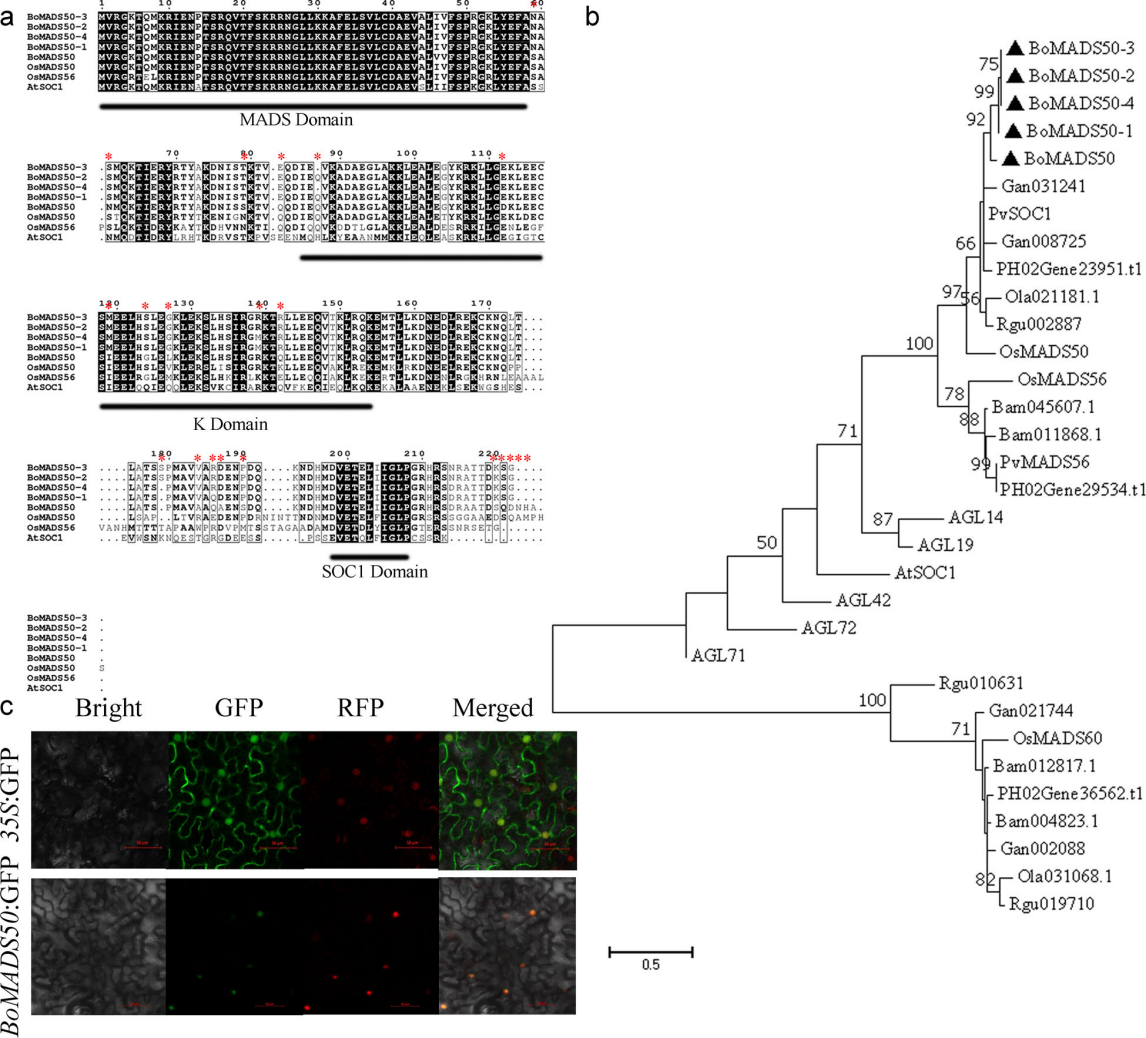
Sequence analysis of SOC1-like proteins in different plant species. **a** The amino acid sequences of SOC1-like proteins were aligned using the ClustalX 2.0 program. Identical and conserved residues are highlighted (black), and the different amino acid site mutations are marked by red stars. **b** A phylogenetic tree of BoMADS50 and SOC1-like proteins from other species was constructed with MEGA 7 using the maximum likelihood (ML) method with 1000 bootstrap replicates based on a multiple sequence alignment result. Bootstrap values higher than 50% are shown on the nodes. Twenty-seven SOC1-like proteins were used: six from *A. thaliana* (AGL14, AGL19, AGL42, AGL71, AGL72, and AtSOC1), three from rice (OsMADS50, OsMADS56, and OsMADS60), three from moso bamboo (PH02Gene29534.t1, PH02Gene23951.t1, and PH02Gene36562.t1), two from *P. violascens* (PvSOC1 and PvMADS56), three from *R. guianensis* (Rgu002887, Rgu019710, and Rgu010631), two from *O. latifolia* (Ola021181.1 and Ola031068.1), four from *G. angustifolia* (Gan021744, Gan002088, Gan031241, and Gan008725) and four from *B. amplexicaulis* (Bam011868.1, Bam045607.1, Bam012817.1, and Bam004823.1). BoMADS50 and BoMADS50-1/2/3/4 are marked by black triangles. **c** The subcellular localization of BoMADS50. *Agrobacteria* carrying BoMADS50 or control vector GFP were infiltrated into leaves of *N. benthamiana* with the nuclear maker *OsART1*-RFP, and the fluorescence images were taken in a dark field for green and red fluorescence, in the white field for the morphology of the cell, and in combination. Bright: bright field; GFP: GFP fluorescence; RFP: RFP fluorescence; Merged: GFP/bright/RFP field overlay. Bar = 50 μm

To isolate the *SOC1*-like gene from *B. oldhamii*, we cloned a cDNA fragment that showed high similarity to *SOC1* orthologs from various plant species. The cDNAs with the full CDS of *BoMADS50* were obtained using the 3′ and 5′ RACE methods. The full-length coding sequence (CDS) region of *BoMADS50* was 678 bp, encoding 225 aa. Similar to those of other known *SOC1*-like genes, the amino acid sequence of *BoMADS50* contained a well-conserved MADS domain, a less conserved K domain, and a divergent C-terminal region (Fig. 1a). Moreover, multiple sequence alignment confirmed the presence of a conserved SOC1 motif that is specific to the TM3-like (tomato MADS-box gene 3-like) clade of *MADS-box* genes in the BoMADS50 protein^22^. BoMADS50 shared high sequence similarities with OsMADS50 in rice and SOC1-like proteins from other bamboo species, further indicating that BoMADS50 is a SOC1 homolog in *B. oldhamii*, which belongs to a SOC1-like group (Fig. 1b). Finally, as shown in Fig. 1c, the fused protein of BoMADS50-GFP was explicitly localized in the nucleus, indicating that *BoMADS50* functions in the nuclei.

### Characteristics of *B. oldhamii* flowering and expression analysis of *BoMADS50*

**Fig. 2:**
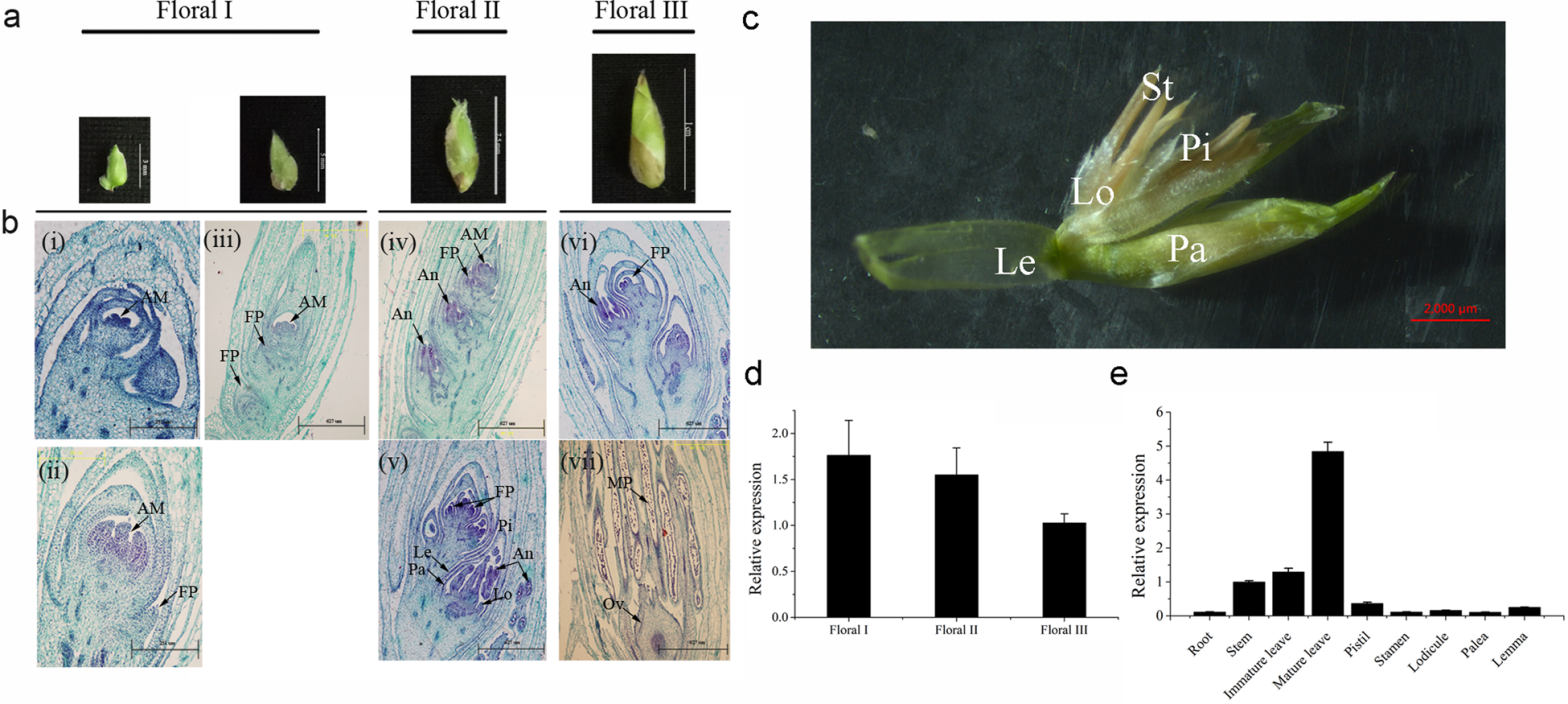
Detection of flowering in *B. oldhamii* and the *BoMADS50* expression pattern. **a** Morphology of *B. oldhamii* inflorescences of different sizes and **b** paraffin sections with scales of 25.1 or 62.7 μm. **c** Morphology of floral organs in a mature flower. **d** and **e** Expression patterns of *BoMADS50* in different vegetative and reproductive tissues. AM apical meristem, FP floret primordium, St stamen, An anther, Pi pistil, Le lemma, Pa palea, Lo lodicule, MP mature pollen, Ov ovule

*B. oldhamii* has a mixed inflorescence with a spikelet as the basic unit^33^. Here, we separated spikelets into three developmental stages based on their size: floral I (~3.00 ± 0.35–4.33 ± 0.50 mm), floral II (~6.69 ± 0.71–7.25 ± 0.50 mm), and floral III (9.57 ± 0.53–11.08 ± 0.36 mm) (Fig. 2a). At the early stage of spikelet differentiation (floral I), the apical meristem has a semiorbicular shape (Fig. 2b-i). This structure continued to elongate and enlarge and produced the protuberance, which developed into the floret primordium (Fig. 2b-ii and iii). In floral II, the floret primordium continued to develop from the base to the top in the spikelet (Fig. 2b-iv). The floret at the base developed first, and then, the flower organ primordium appeared and gradually developed into the lemma, palea, lodicule, stamen, and pistil (Fig. 2b-v). At the last stage (floral III), the flower bud primordium at the top began to differentiate (Fig. 2b-vi). All the flower organs in the basal floret completed development and formed mature pollen grains and ovaries (Fig. 2b-vii). Thus, the floral primordium of *B. oldhamii* was formed as early as in the floral I stage and kept forming from the base to the top in the spikelet. Mature flower organs gradually appeared in the base spikelet (floral II). When the top floret matured, the development of the spikelet ended (floral III).

The mature flowers of *B. oldhamii* had six stamens, one pistil, three lodicules, one palea, and one lemma (Fig. 2c). However, even when the flora matured, the stigma usually failed to be exposed, and the stamens were mostly withered (Fig. S1a). Many wrinkled and defective pollens formed, and two layers of anther walls were observed (Fig. S1b). These flowering characteristics and abnormal microspores are likely the main reasons for the sterility of *B. oldhamii*^34^.

To understand the putative function of *BoMADS50* in flower development, we examined the expression pattern of *BoMADS50* in various vegetative and reproductive organs of *B. oldhamii*. As shown in Fig. 2d, during in vitro flowering, *BoMADS50* expression reached its highest level at the floral I stage, decreased gradually with the development of spikelets, and was lowest in the floral III stage. In the vegetative tissues, *BoMADS50* was highly expressed in mature leaves, which was more than five times higher than that in roots and stems (Fig. 2e). In flower organs, the expression of *BoMADS50* was barely detected. This finding indicates that *BoMADS50* may integrate flowering signals from the leaves for the transition from a vegetative to a reproductive state but does not directly regulate flower organ development.

### Overexpression of *BoMADS50* accelerates flowering in rice

**Fig. 3:**
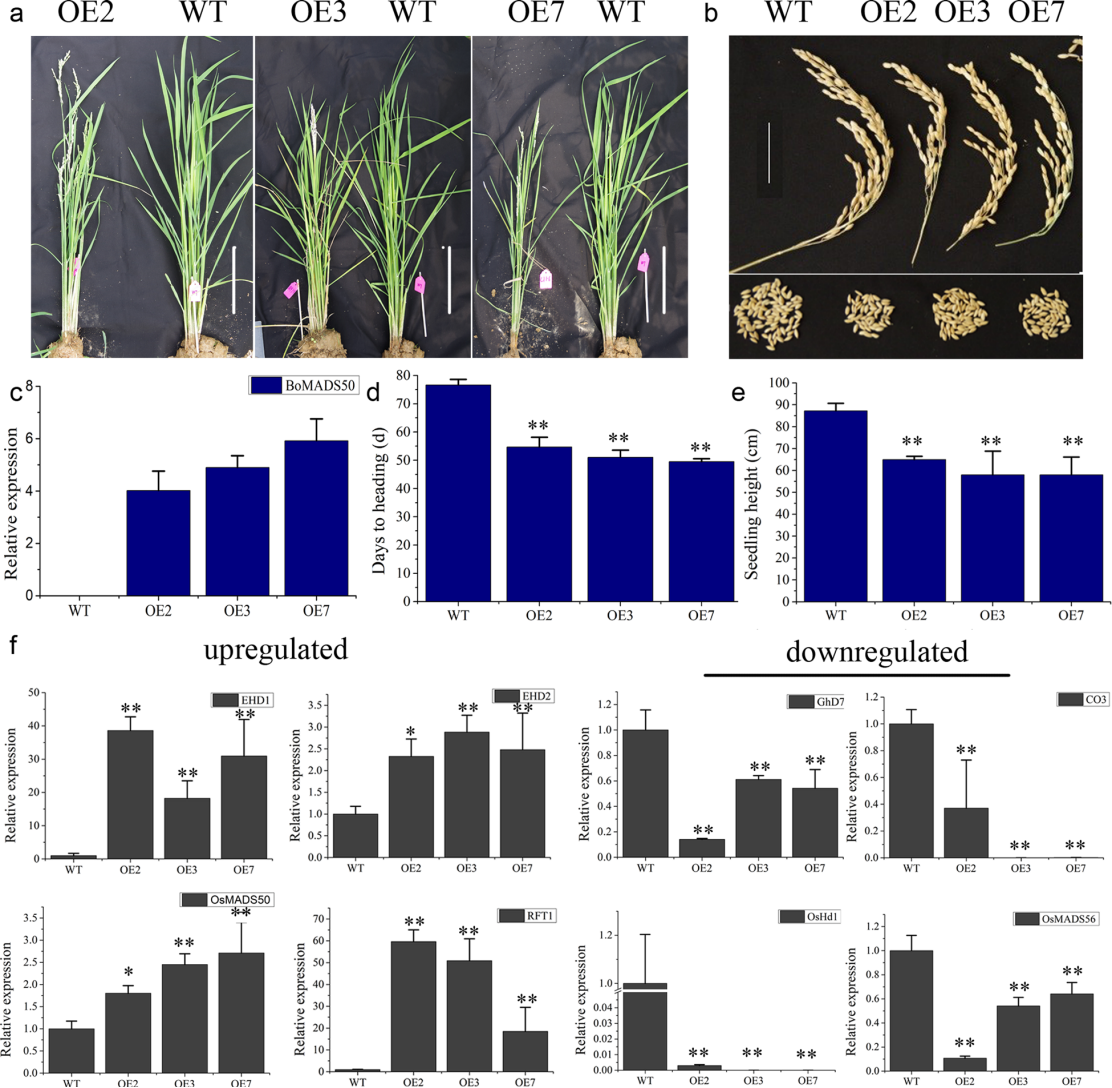
Overexpression of *BoMADS50* promotes flowering in rice. **a** Morphology of rice plants overexpressing *BoMADS50* at flowering. Bar = 10 cm. **b** Morphology of seeds of *BoMADS50*-overexpressing rice. **c** Expression level of *BoMADS50* in three independent lines. **d** Heading days and (**e**) height of three *BoMADS50*-overexpressing lines. All data are the mean ± s.d. (*n* ≥ 10 independent plants for each line). Asterisks indicate significantly different values (**P < 0.01). **f** Analysis of mRNA abundance of flowering-related genes in transgenic rice lines and WT. Asterisks indicate that the value is significantly different from that of the WT at the same time point (*P < 0.05, **P < 0.01)

As bamboo has a closer relationship with rice, we transformed *BoMADS50* into rice to determine its role in the regulation of flowering (Fig. 3). Three independent homozygous lines of *BoMADS50* were selected (*OE2/3/7*, grown under field conditions) according to different gene expression levels in the T3 generation. Compared with wild-type (WT) plants, the *BoMADS50* overexpression plants showed phenotypes such as earlier flowering, smaller panicles, and fewer seeds (Fig. 3a, b). In transgenic rice, the average relative expression level of *BoMADS50* was ~4.95 ± 0.95 (Fig. 3c), and the heading time and seedling height were ~51.74 ± 2.65 days and 60.33 ± 4.04 cm, respectively, ~25 days earlier and 27 cm shorter than those of the WT lines (76.62 ± 2.02 days and 87.22 ± 3.39 cm) (Fig. 3d, e). To study the molecular mechanism by which *BoMADS50* promotes flowering, we analyzed the expression patterns of the critical regulators in the rice photoperiod pathway (Fig. 3f). In the *BoMADS50*-overexpressing plants, the expression of *Early heading date2* (*Ehd2*), *OsMADS50*, *RFT1,* and *Ehd1* was obviously upregulated, approximately 2.56 ± 0.28, 2.32 ± 0.46, 42.99 ± 21.68, and 29.27 ± 10.29 times higher than that in the WT plants, respectively. In addition, the expression of *OsHd1*, *OsCO3* (*a CONSTANS-LIKE gene*), *Ghd7* (a *CO*-like gene containing a CCT motif), and *OsMADS56* was clearly downregulated after overexpression of *BoMADS50*, leading to a decrease of at least ~60%. Therefore, ectopic expression of *BoMADS50* can promote the flowering of transgenic rice. Further, for confirmation of its role in flowering, *BoMADS50* was ectopically overexpressed in the *soc1* mutant and WT *A. thaliana*. As shown in Fig. S2, overexpression of *BoMADS50* rescued the late-flowering phenotype of *soc1* mutants (Fig. S2a–c) and accelerated flowering in transgenic *A. thaliana* by regulating downstream flowering genes (Fig. S2d–h). Thus, the above results indicate a positive role of *BoMADS50* in promoting flowering.

### BoMADS50 interacts with AP1/FUL-like proteins in *B. oldhamii*

**Fig. 4:**
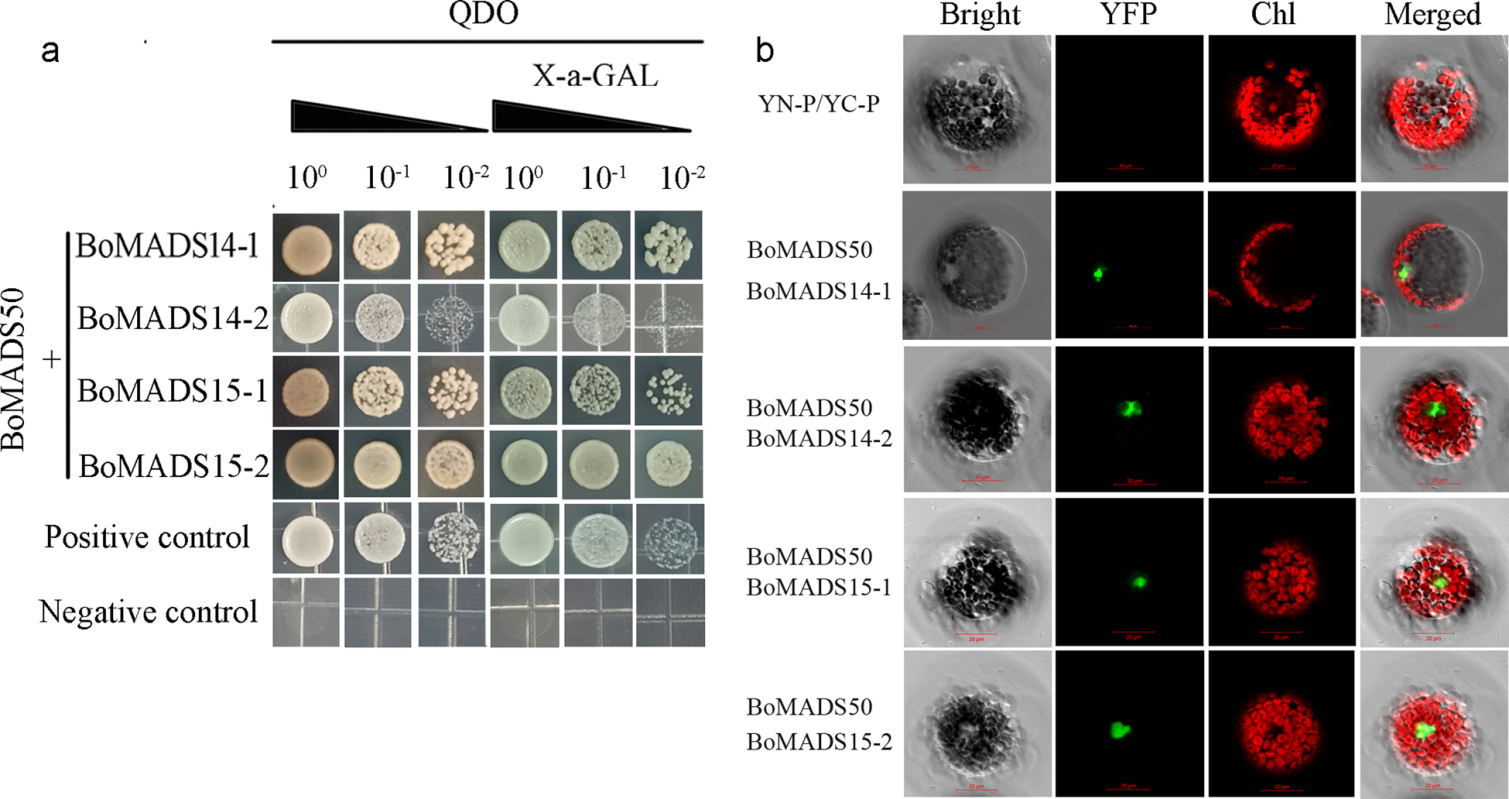
Protein–protein interaction of BoMADS50 and AP/FUL-like proteins by yeast two hybridization (Y2H) and bimolecular fluorescence complementation (BiFC) assays. (**a**) and (**b**) Show that BoMADS50 could form dimers with BoMADS14-1, BoMADS14-2, BoMADS15-1, and BoMADS15-2 in both yeast cells and *A. thaliana* protoplasts. In (**a**), pGBKT7-53 and pGADT7-T, pGBKT7-Lam, and pGADT7-T were used as positive controls and negative controls, respectively. In (**b**), the empty vectors were used as negative controls. The cotransformation results of BoMADS50, BoMADS14-1, BoMADS14-2, BoMADS15-1, and BoMADS15-2 with the YN-P or YC-P vector are shown in Fig. S4. Scale bar = 20 μm. Bright bright field, YFP YFP fluorescence, Chl chlorophyll autofluorescence, Merged YFP/bright/Chl field overlay

The *APETALA1/FRUITFULL* (*AP1/FUL*)*-like* genes determine flower meristem characteristics and are key genes for flowering induction and morphology^18,19^. Here, four homologs of *OsMADS14* and *OsMADS15* were identified and obtained from *B. oldhamii* (*BoMADS14-1*, *BoMADS14-2*, and *BoMADS15-1*, *BoMADS15-2*) (Fig. S3a). These genes were highly expressed in the floral II or floral III stage, except for *BoMADS14-1*, which showed an expression peak in floral I, similar to *BoMADS50* (Fig. S3b). This finding suggests that *AP1/FUL-like* genes may participate in both floret primordium specification and the flower organ development process in *B. oldhamii*. To test whether BoMADS50 and AP1/FUL proteins interact, we performed yeast two-hybrid (Y2H) experiments. The yeast cells cotransformed with BoMADS50 and BoMADS14-1/2, BoMADS15-1/2, and the positive control grew normally and developed a blue color on -QDO/X medium, whereas those with the negative control did not (Fig. 4a). These results suggest that BoMADS50 can form complexes with the four AP1/FUL-like proteins in yeast cells. Bimolecular fluorescence complementation (BiFC) assay was performed in *A. thaliana* mesophyll protoplast transient expression systems to verify the Y2H results (Fig. 4b). Except for the negative controls (Figs. 4b and S4), green fluorescence was observed in *A. thaliana* protoplasts cotransformed with vectors containing BoMADS50 and BoMADS14-1/2 and BoMADS50 and BoMADS15-1/2. Therefore, both the Y2H and BiFC assays confirmed the ability of BoMADS50 to heterodimerize with the BoMADS14-1, BoMADS14-2, BoMADS15-1, and BoMADS15-2 proteins in vivo.

### BoMADS14-1 can bind to the *BoMADS50* promoter and activate its transcription

**Fig. 5:**
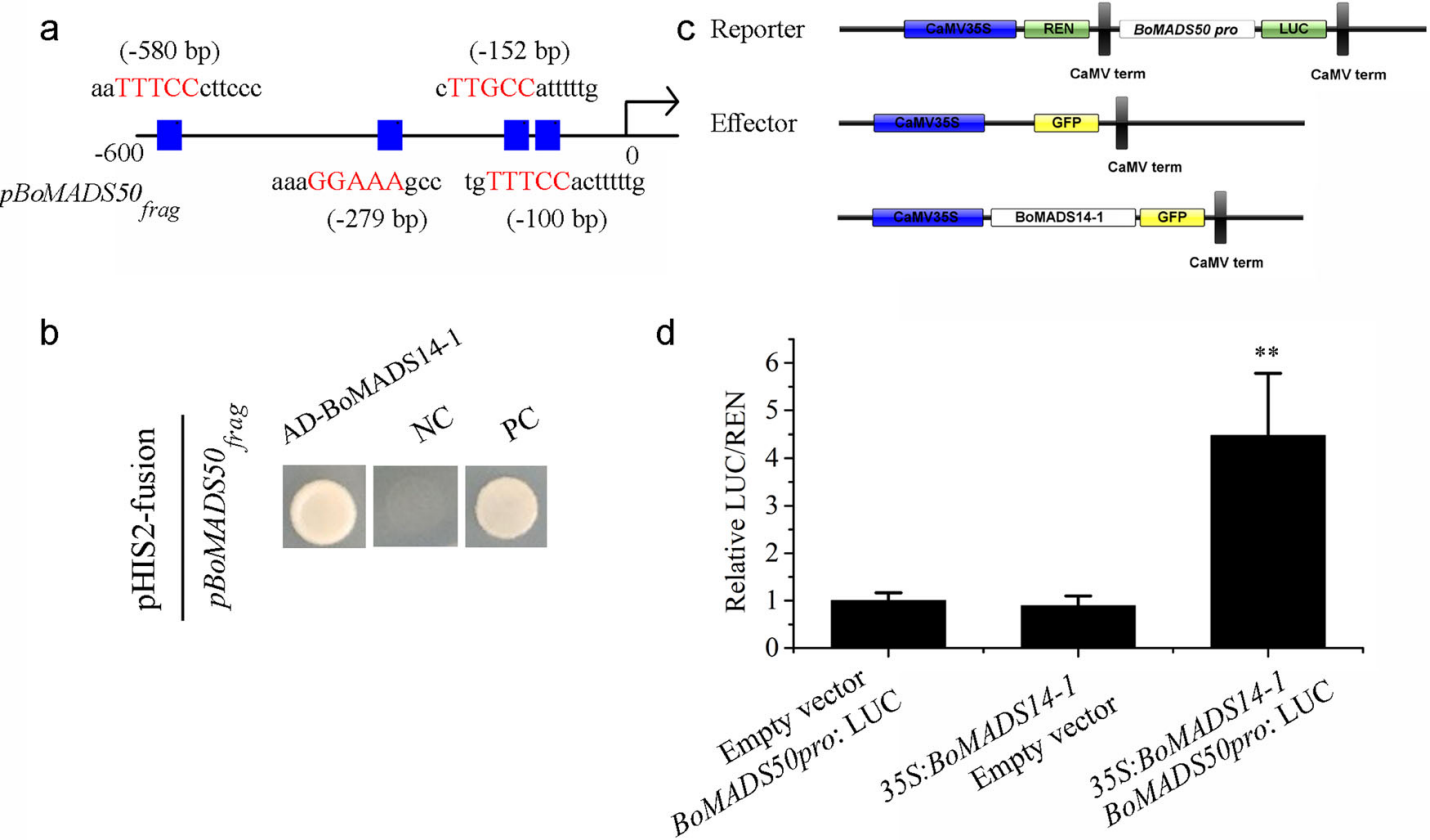
BoMADS14-1 promotes *BoMADS50* expression. **a** Diagram of the *BoMADS50* promoter region containing the putative CArG motif. **b** BoMADS14-1 bound to the *BoMADS50* promoter regions with the putative CArG motif and activated the expression of the nutritional reporter gene HIS2 by yeast one-hybrid assays. **c** Effector and reporter constructs used in transient dual-luciferase assays. **d** BoMADS14-1 triggered the expression of *BoMAD50pro:*LUC with the integration of the 2000-bp *BoMADS50* genomic sequence upstream of the ATG before LUC. All data are the mean ± s.d. (*n* ≥ 3). Asterisks indicate significantly different values (**P < 0.01)

Given the similar expression patterns of *BoMADS50* and *BoMADS14-1* and the putative role of *AP1* in activating flowering, we speculated that *BoMADS14-1* might be involved in *BoMADS50* expression regulation. The promoter of *BoMADS50* was cloned and analyzed using the PlantPAN 3.0 database, identifying four CArG motifs located in different clusters (Fig. 5a). The binding affinity of BoMADS14-1 for the *BoMADS50* promoter was tested using a yeast one-hybrid (Y1H) assay with a 489 bp fragment, and BoMADS14-1 was shown to bind to the fragment containing a putative CArG motif and activate HIS2 nutritional reporter gene expression (Fig. 5b). Furthermore, we carried out a transient assay to analyze the activating or repressive effect of BoMADS14-1 on the expression of *BoMADS50*_*pro*_*:*LUC containing a complete promoter (Fig. 5c). Compared with the coexpression of empty vector as the negative control, that of the fusion protein of BoMADS14-1 triggered and significantly upregulated the expression of *BoMADS50*_*pro*_*:*LUC (Fig. 5d). The above results suggest that BoMADS14-1 can positively regulate *BoMADS50* expression in vivo.

### BoMADS50 and BoMADS50-1 have different regulatory activities

Our study identified four additional transcripts of *BoMADS50* (*BoMADS50-1/2/3/4*), showing different nucleotide variations. Compared with BoMADS50, BoMADS50-1/2/3/4 had shorter C-terminal regions and various amino acid mutations (Fig. 1a). These sequences belong to an OsMADS50 subgroup and showed high similarities with other *OsMADS50*-like genes in different bamboo species (Fig. 1b). Ectopic transformation analysis showed that overexpression of *BoMADS50-1/2/3/4* could restore the late-flowering phenotypes of *soc1 A. thaliana* mutants (Fig. S2). Moreover, overexpression of *BoMADS50-1/2/3/4* accelerated the flowering of transgenic rice (grown in a greenhouse, Fig. S5), leading to an ~15-day reduction in flowering time compared with that of the WT (Table S2). Thus, *BoMADS50-1/2/3/4* acts as flowering activators.

**Fig. 6:**
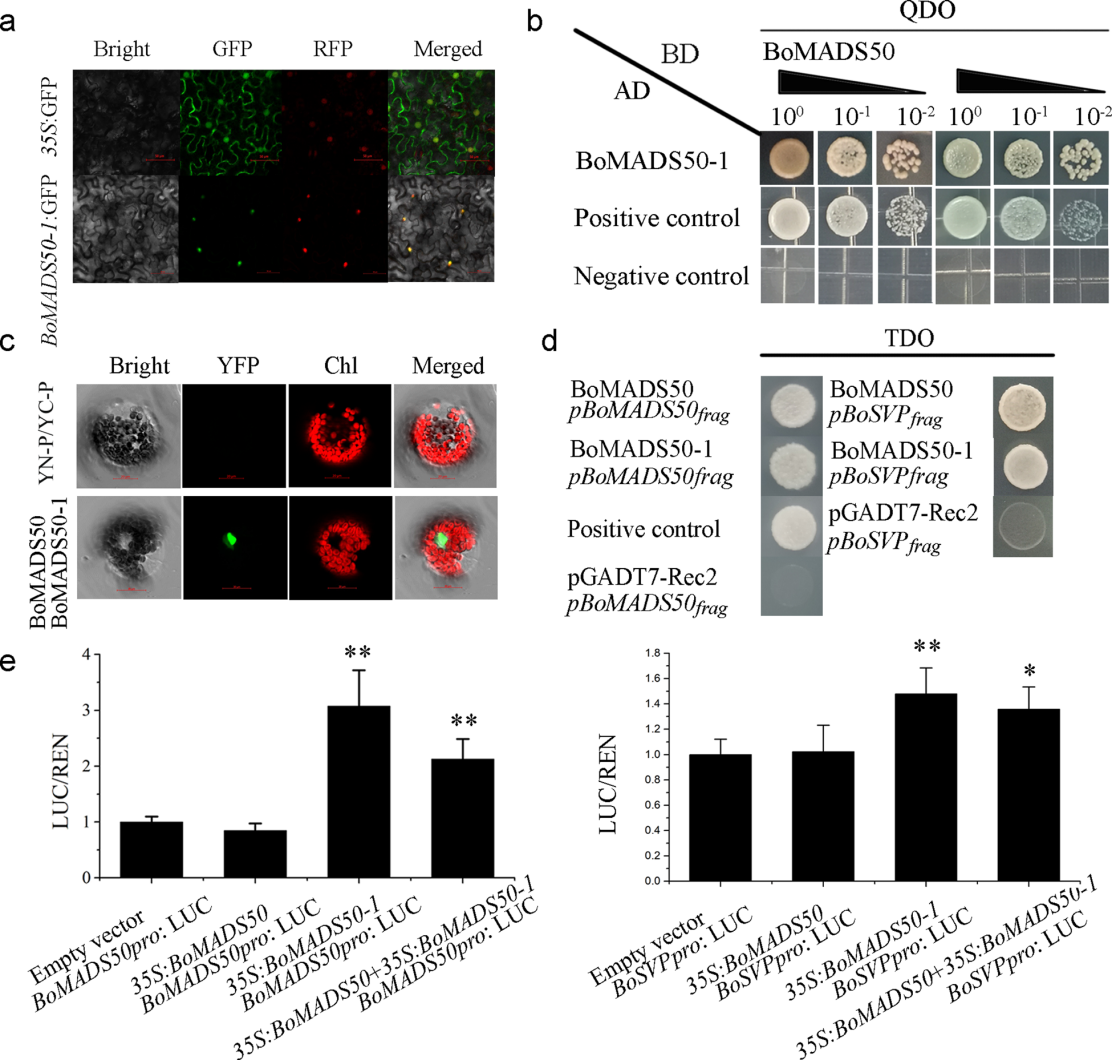
BoMADS50-1 can interact with BoMADS50 and promote its own expression and *BoSVP*. **a** The subcellular localization of BoMADS50-1. *Agrobacteria* carrying BoMADS50-1 or control vector GFP were infiltrated into leaves of *N. benthamiana* with the nuclear maker *OsART1*-RFP, and the fluorescence images were taken in dark fields for green and red fluorescence, in the white field for the morphology of the cell, and in combination. Scale bar = 50 μm. Bright: bright field, GFP: GFP fluorescence, RFP: RFP fluorescence, Merged: GFP/bright/RFP field overlay. **b** and **c** BoMADS50-1 could form dimers with BoMADS50 in both yeast cells and *Arabidopsis* protoplasts. Scale bar = 20 μm. Bright: bright field, YFP:YFP fluorescence. Chl: chlorophyll autofluorescence, Merged: YFP/bright/Chl field overlay. **d** Both BoMADS50 and BoMADS50-1 bound to the *BoMADS50* and *BoSVP* promoter regions with putative CArG motifs and activated the expression of the nutritional reporter gene *HIS2* in yeast one-hybrid assays. **e** BoMADS50-1 triggered the expression of *BoMAD50pro*:LUC and *BoSVPpro*:LUC by integration of 2000-bp *BoMADS50* and *BoSVP* genomic sequences upstream of the ATG before LUC. The cotransformation of BoMADS50 decreased the binding ability of BoMADS50-1 to itself and *BoSVP*. All data are the mean ± s.d. (*n* ≥ 3). Asterisks indicate significantly different values (**P < 0.01)

To determine whether there is functional diversification among different transcripts, we selected *BoMADS50-1* for further investigation. BoMADS50-1 was also localized in the nucleus (Fig. 6a). Both Y2H and BiFC assays showed that BoMADS50-1 could interact with BoMADS50 and form a heterodimer in vivo (Fig. 6b, c). In bamboo, the *SVP*-like gene can promote flowering in transgenic rice^35^, and in this study, we found that *BoSVP* has an expression pattern similar to that of *BoMADS50* (Fig. S6). This finding indicates that *BoSVP* functions upstream or downstream of *BoMADS50*. Y1H results showed that both BoMADS50 and BoMADS50-1 could bind promoter fragments of itself and *BoSVP* (Fig. 6d). However, in the dual-luciferase reporter assays, only *BoMADS50-1* significantly promoted *BoMADS50* and *BoSVP* expression (Fig. 6e). Moreover, the LUC/REN activity was decreased when *BoMADS50-1* and *BoMADS50* were cotransformed with target sequences. This result suggests that *BoMADS50* could reduce the binding ability of *BoMADS50-1* to itself and downstream targets. Thus, we propose that *BoMADS50* and *BoMADS50-1* have different regulatory activities.

## Discussion

### *BoMADS50* acts as a flowering promoter in transgenic *A. thaliana* or rice

Woody bamboos typically exhibit gregarious flowering cycles up to 120 years followed by the death of the parent plants, unique among flowering plants. Although many hypotheses about the bamboo flowering cycle have emerged^3,4,9^ and the functions of some flowering genes have been preliminarily analyzed^36–38^, “the molecular aspects of bamboo flowering still remain at a nascent stage”^14^. The SOC1 protein is an essential integrator of multiple flowering signals, regulating the flowering time, floral patterns, and even floral meristem determinacy^24,39,40^. As described by Guo et al.^2^, *SOC1/OsMADS50* orthologs in woody bamboos may be malfunctioning because of protein structure changes or insertions of transposable elements. These malfunctions are the potential cause of the extremely long vegetative growth phase in woody bamboos. In this study, a *SOC1/OsMADS50-like* gene, *BoMADS50*, was identified from *B. oldhamii*. However, the MADS-box domain of *BoMADS50* was shown to be complete, although a 179 bp interspersed repeat element was inserted in the ORF region of *BoMADS50* (Table S3). We further verified the functional role of BoMADS50 by ectopic expression in rice and *A. thaliana*. Similar to *PvSOC1* from *P. violascens*^32^ and other *SOC1*-like genes^25,41,42^, *BoMADS50* overexpression accelerates flowering in transgenic rice and *A. thaliana* and can rescue the late-flowering phenotype of *soc1* mutants (Figs. 3 and S2). In rice, *OsMADS50* is an upstream regulator of *Ehd1* and *RFT1* that regulates flowering under LD conditions^28,29^ but functions either parallel to or downstream of *Hd1*, *Ghd7*, and *Ehd2*^28,43,44^. Here, transcript levels of *Ehd1* and *RFT1* were both upregulated significantly in the *BoMADS50-*overexpressing rice (Fig. 3), indicating that *BoMADS50* is involved in an *Ehd1–RFT1* pathway to regulate flowering, which is similar to *OsMADS50*. Interestingly, *BoMADS50* overexpression also significantly induced the expression of *Ehd2* but repressed that of *Hd1* or *Ghd7* (Fig. 3). This finding suggests that *BoMADS50* may be involved in multiple pathways (*OsHd1–OsHd3a/RFT1*, *Ghd7–Ehd1,* and *Edh2–Ehd1*) to affect flowering time in transgenic rice^29^. Thus, in contrast to the previously suggested idea of Guo et al.^2^, *BoMADS50* acts as a flowering promoter, and there is no obvious loss or malfunction of *BoMADS50* in flowering regulation, at least as shown in transgenic rice or the *A. thaliana* system.

### *BoMADS50* may be involved in the flowering regulation of *B. oldhamii*

In rice and most species, *SOC1*-like genes are highly expressed in mature leaves, suggesting their role in the transition from the vegetative to the reproductive phase^32,41,43,45,46^. Moreover, in *E. japonica*, the expression level of *EjSOC1* in early flower buds was significantly higher than that in blooming flowers^27^. Here, expression analysis results showed that *BoMADS50* was highly expressed in the floral I stage and mature leaves of *B. oldhamii* (Fig. 2), indicating a potential role of *BoMADS50* in flower primordium formation and flowering initiation. However, highly expressed *BoMADS50* in the *B. oldhamii* mature leaves does not induce flowering in this species. This finding may be because the expression of *BoMADS50* has not reached the threshold level that can lead to floral initiation. In addition, plant flowering is regulated by a complex gene network^29,39,47^. High expression of *BoMADS50* alone may not be sufficient to induce *B. oldhamii* flowering.

SOC1-like protein is known as an active player by interacting with many other MADS proteins^23,24,48^. For example, SOC1 and AP1/FUL-like proteins can form a network during flowering regulation, and AP1/FUL proteins act downstream or as repressors of *SOC1*-like genes in model plants^24,39,43,47^. *OsMADS14* and *OsMADS15* are key AP1/FUL factors involved in flower development and floral initiation regulation in rice^18,19,49^. In this study, the *OsMADS14* homologs *BoMADS14-1* and *BoMADS14-2* and the *OsMADS15* homologs *BoMADS15-1* and *BoMAD15-2* were highly expressed in different flower development stages (Fig. S3), indicating their potential regulatory roles in floral induction and floral meristem determination of *B. oldhamii*. We also found that BoMADS50 can interact with BoMADS14-1/-2 and BoMADS15-1/-2 in both yeast and plant cells, but *BoMADS14-1* functioned upstream of *BoMADS50* by significantly activating its expression (Figs. 4 and 5). Thus, BoMADS50 probably participates in regulating flowering induction by acts synergistically with BoAP1/FUL proteins, and a diversified regulatory relationship between BoMADS50 and BoAP1/FUL might exist in *B. oldhamii*. The results of heterologous transformation, expression profile, and protein network analysis all imply that *BoMADS50* maybe a flowering regulator in *B. oldhamii*; however, there are still some considerations. The most important reason is that gene functions in a heterologous system may differ from those in the original species. For example, Voogd et al.^42^ showed that overexpression of kiwifruit *SOC1*-like genes did not cause early flowering in kiwifruit, although their overexpression did in *A. thaliana*. Therefore, future studies should focus on establishing the exact role of *BoMADS50* in transgenic bamboo.

### The functional conservatism and specificity of *BoMADS50* and B*oMADS50-1* might be related to the synchronized and sporadic flowering characteristics of *B. oldhamii*

In this study, four additional transcripts (*BoMADS50-1/2/3/4*) of *BoMADS50* were identified. They were clustered with *BoMADS50* together in our phylogenetic tree, indicating close evolutionary relationships among them. As shown in a previous study, *B. oldhamii* belongs to the paleotropical (PTB) woody bamboos^50^, suggesting that multiple copies of the *OsMADS50* ortholog might exist in *B. oldhamii*^2^. However, the ploidy level of *B. oldhamii* has not been determined. Therefore, whether *BoMADS50* and *BoMADS50-1/2/3/4* are alleles or homeologs has not been confirmed. *BoMADS50-1/2/3/4* showed polymorphisms in protein-CDS compared to *BoMADS50*, but ectopic expression results suggest their conservative roles in promoting flowering (Fig. S5). Interestingly, we found that only *BoMADS50-1* could induce the expression of itself and *BoSVP* (Fig. 6), indicating a different regulatory ability of *BoMADS50* and *BoMADS50-1*. Protein structure analysis showed that BoMADS50-1 had a coding frameshift at the C-terminus due to base variations (Fig. 1). For the full function of MADS proteins, the C-terminal motifs are essential and may be responsible for the functional diversification of the major MADS gene subfamilies^22^. Therefore, we speculate that nucleotide variations might endow *BoMADS50-1* with a stronger regulatory ability than *BoMADS50* by changing the C-terminal structure.

There are many theories about flowering regulation in bamboo^3,4,9^, but many questions remain unanswered. For example, the average flowering time is ~30–60 years for *B. oldhamii*, but in the early periods of bamboo life, some clumps or small areas of scattered bamboo will blossom sporadically within a *B. oldhamii* population^4,8^. Franklin^3^ suggested that the term “sporadic flowering” may imply random or other nongregarious patterns of flowering, but there is no convincing evidence that any semelparous bamboo has a reproductive strategy that may be regarded as “not gregarious”. Zheng et al.^4^ noted that sporadic flowering or partial flowering may be due to the bamboo forest being composed of different clones, thus forming a flowering wave. However, in *Sasa cernua* and *P. violascens*, flowering clumps and nonflowering vegetative clumps were discovered to be of the same clone^51,52^. Therefore, some mechanistic malfunctions or physiological connections probably influence the sporadic flowering of bamboo^9^.

In many species, single-nucleotide polymorphisms (SNPs) are known to be associated with different flowering time genotypes^53,54^. These identifications led us to speculate that the variation in regulatory ability between *BoMADS50* and *BoMADS50-1* caused by nucleotide variations may be related to the synchronized and sporadic flowering characteristics of *B. oldhamii*. For example, in some flowering clumps, *BoMADS50-1* with more robust activity might be selected as a major gene form, leading to different expression levels of downstream flowering genes compared to those of *BoMADS50* and resulting in different flowering times. For other vital flowering genes, there may also be different transcripts. The functional conservation of these transcripts could ensure synchronized flowering within a bamboo population, while their potentially different regulatory abilities may cause the sporadic flowering phenotype. It is still challenging to obtain the quantitative loci associated with flowering time due to the unique flowering characteristics of bamboo. To some extent, our results provide a possible hypothesis for the combined extensive synchronized and sporadic flowering phenomenon of woody bamboos.

In summary, based on our results and previous studies in model plants, we propose a hypothetical working model of *BoMADS50* in regulating flowering. In addition, *BoMADS50-1* was revealed to have a stronger activation ability than *BoMADS50*, indicating that nucleotide variations may lead to changes in regulatory activity. Above all, *BoMADS50 is* probably an important flowering regulator in bamboo. Further, the functional conservatism and specificity of *BoMADS50* and *BoMADS50-1* may be related to the synchronized and sporadic flowering characteristics in *B. oldhamii*.

## Materials and methods

### Plant materials and growth conditions

The flowering tissue culture system is important for woody bamboo flowering mechanistic studies^55^. For *B. oldhamii*, the flowering buds and vegetative shoots originated from the same tissue culture strains^56^. In this study, inflorescences of different developmental periods and different reproductive organs (including pistils, stamens, lemmas, paleas, and lodicules) were derived from these flowered tissue culture plants and have continued flowering since 2001. Moreover, vegetative organs (including roots, stems, immature leaves, and mature leaves) were derived from nonflowering tissue culture plants that had never flowered since their initial culture in 1991^8^. All plants were grown under a 16-h light/8-h dark photoperiod indoors at a temperature of 25–27 °C.

### Gene identification and cloning

cDNA from flower buds in different culture tissue lines in *B. oldhamii* was used as a templet for gene cloning. To isolate a *SOC1-*like gene from *B. oldhamii*, we compared the amino acid sequences of *SOC1* homologs from grass family plants, including rice, *Triticum aestivum,* and *Z. mays*, and designed primers (S-F/R) for conserved domain regions. A specific *SOC1-*like cDNA fragment (~250 bp) was obtained, ligated into the pMD18-T vector and confirmed as a partial sequence of a *SOC1-*like gene of *B. oldhamii*. The 5′ and 3′ RACE systems were used to isolate the 5′ and 3′ end cDNA with the Rapid Amplification of cDNA Ends kit (Invitrogen, USA). A 330 bp 5′-RACE cDNA and 740 bp 3′-RACE cDNA fragment were spliced to obtain 1049 bp full-length cDNA. With this sequence as the template, the ORF of *BoMADS50* was amplified with a high-fidelity enzyme (TaKaRa, Japan). Moreover, four transcripts of *BoMADS50* were obtained from *B. oldhamii*, named *BoMADS50-1*, *BoMADS50-2*, *BoMADS50-3*, and *BoMADS50-4*. Finally, the nucleotide variations were further confirmed by PCR using DNA templates of different lines. The primers used for *BoMADS50* cloning are shown in Table S1.

### Sequence alignment and phylogenetic analysis

For phylogenetic analysis, the SOC1- or AP1-like proteins in rice or *A. thaliana* were downloaded from the Rice Genome Annotation Project database (http://rice.plantbiology.msu.edu/) or The Arabidopsis Information Resource database (https://www.arabidopsis.org/), respectively. For identification of SOC1- or AP1-like proteins in different bamboo species, the amino acid sequences of OsMADS50, OsMADS56, and OsMADS60 (SOC1-like), OsMADS14, OsMADS15, OsMADS18, and OsMADS20 (AP1-like) from rice were used as query sequences to blast against the moso bamboo protein V2 database^11^, *Bonia amplexicaulis*, *Olyra latifolia*, *Guadua angustifolia*, *Raddia guianensi* protein databases^2^ and *B. edulis* database^57^ with an *e*-value cutoff of 10^−50^. Additionally, the amino acid sequence of PvSOC1 in *P. violascens* was obtained from Ma^58^, and the GenBank accession number of PvMADS56 is AQS27937.1^31^. Multiple sequence alignment was performed using ClustalX 2.0, and the phylogenetic tree was constructed with MEGA 7.0 using the maximum-likelihood (ML) method with 1000 bootstrap replicates.

### Expression profile revealed by qRT-PCR

Total RNA was extracted from flowers in bamboo, leaves in transgenic *A. thaliana*, and rice using TRIzol reagent. Single-stranded DNA was synthesized using the PrimeScript™ RT reagent Kit with gDNA Eraser (TaKaRa) according to the instructions. The transcripts of *BoMADS50*, *BoMADS14-1*, *BoMADS14-2*, *BoMADS15-1*, and *BoMADS15-2* in bamboo and the key flowering genes in transgenic plants were assessed using qRT-PCR with specific primers. In *B. oldhamii*, *NTB* was used as a reference gene^59^, while in *A. thaliana* and rice, *Atactin* or *OsUbq* was used as a reference gene. The primers used for gene expression analysis are listed in Table S1. qPCR was performed using SYBR Green Master Mix (Bio-Rad) on a real-time PCR instrument (Bio-Rad). All qPCR assays were performed with three biological and four technical replicates, and the 2^−∆∆CT^ method was used for quantitative analysis. For *BoMADS50* in transgenic rice or *A. thaliana* lines, the expression of the endogenous homologous genes *OsMADS50* or *SOC1* was normalized to 1. For other flowering genes in rice and *A. thaliana*, their relative expression in the WT was normalized to 1.

### Subcellular localization

For the subcellular localization assay, the CDSs of *BoMADS50* and *BoMADS50-1* were cloned into the pCAMBIA-1300-GFP vector to generate *35S:BoMADS50*:GFP and *35S:BoMADS50-1*:GFP. The empty vector was transformed as a negative control, and nuclear-localized OsART1 served as a nuclear localization marker by inducing each transfection^60^. Transient transformation of tobacco leaves was performed as described by Yang et al.^61^. The transformed plants were then cultured in the dark for more than 48 h, followed by GFP fluorescence detection under a confocal microscope (Zeiss, LSM 880) with both 488 and 594 nm argon lasers.

### Yeast two-hybrid (Y2H) assay

The CDS of *BoMADS50* was cloned into the pGBKT-7 vector (Clontech, Beijing, China), and the CDSs of putative interacting genes (*BoMADS50-1*, *BoMADS14-1*, *BoMADS14-2*, *BoMADS15-1* and *BoMADS15-2*) were cloned into pGADT7 vectors. pGBKT7-BoMADS50 was cotransformed with the corresponding pGADT7 recombinant plasmid into the yeast strain AH109 following the manufacturer’s instructions for the Matchmaker GAL4 two-hybrid system (Clontech). pGBKT-53+pGADT7-T and pGBKT53+pGADT-Lam were used as positive and negative controls, respectively. The transformed yeast was cultured in SD/-Trp/-Leu (-DDO) media at 30 °C for 3 days, and a serial decimal dilution was used for the spot assay on dropout media SD/-Trp/-Leu/-His/-Ade (-QDO) and -QDO/X-a-Gal (-QDO/X). The protein interactions were confirmed for ~3–5 days at 30 °C.

### BiFC assay

The CDS of *BoMADS50* was cloned into the pSAT1-nEYFP-C1 (YN-P) vector. The other genes of interest were cloned into the pSAT4-cEYFP-C1-B (YC-P) vector. All empty vectors were used as a negative control, while the recombinant plasmids (YN-*BoMADS50*, *BoMADS50-1*-YC, *BoMADS14-1*-YC, *BoMADS14-2*-YC, *BoMADS15-1*-YC, *BoMADS15-2*-YC) were also cotransformed with the YN-P or YC-P vector to exclude false positives. These constructs were transiently expressed in *A. thaliana* mesophyll protoplasts, according to a previous report^62^. The transfected cells were imaged using a confocal microscope at wavelengths of 488 nm and a 594 nm argon laser. The combinations of BiFC were performed in at least three biological replicates.

### Y1H assay

We cloned the CDSs of *BoMADS50*, *BoMADS50-1*, and *BoMADS14-1* into the vector pGADT7-Rec and the 489-bp *BoMADS50* promoter fragment and 416-bp *BoSVP* promoter fragment containing the putative CArG motif into the vector pHIS2. The yeast strain Y187 was cotransformed with the corresponding pGADT7 and pHIS2 constructs following the manufacturer’s instructions for the yeast one-hybrid library screening system (Clontech). pHIS53 + pGADT753 was used as a positive control, and pHIS-*proBoMADS50*_frag_ + pGADT7-Rec and pHIS-*proBoSVP*_frag_ + pGADT7-Rec were used as negative controls. The transformed strain was cultured at 30 °C for 2–3 days on SD/-Trp/-Leu media, and serial decimal dilution was used for spot assays on SD/-Trp/-Leu/-His media with 50 mM 3-AT.

### Dual-luciferase reporter assay

The *BoMADS50* and *BoSVP* promoters (2000 bp genomic sequence before ATG) were amplified and cloned into the pGreenII 0800-LUC vector, which generated the reporter construct *BoMADS50pro*:LUC or *BoSVPpro*:LUC. The effectors were constructed by cloning the open-reading frames into the pCAMBIA1300 vector with Flag driven by the CaMV 35S promoter (*35S:BoMADS50*, *35S:BoMADS50-1*, *35S:BoMADS14-1*). The empty vectors were coexpressed with reporter or effector as a negative control. The transformation of all the effectors and reporters followed the protocol of^16^. *Agrobacterium tumefaciens* was cultured overnight and resuspended to OD_600_ values of 0.6–0.8. The mixture with the effector and reporter strain at a ratio of 9:1 was resuspended in infiltration media (10 mM MES, 1 M MgCl_2_, 200 μM acetosyringone) and incubated for 3–4 h at room temperature without shaking. The infiltrated young *Nicotiana benthamiana* plants were incubated for more than 48 h under weak light conditions. At least four biological replicates were performed for each sample. The LUC/REN signals were calculated using dual-LUC assay solutions (Promega) on a luminometer (Promega). Finally, the LUC/REN value of the control group (coexpressed with empty vector and *BoMADS50pro*:LUC; empty vector and *BoSVPpro*:LUC) was normalized to 1.

### Vector construction and generation of *BoMADS50*-overexpressing *A. thaliana* and rice

The recombinant plasmids *pCAMBIA1300-BoMADS50* and *pCAMBIA1300-BoMADS50-1/2/3/4* were introduced into *A. tumefaciens* GV3101 and EHA105, respectively. The floral dip method was used for *A. thaliana* transformation, and an *Agrobacterium*-mediated rice transformation method was described by Nishimura et al.^63^. Regenerated seedlings were selected on MS medium with 50 mg L^−1^ hygromycin. The expression levels of *BoMADS50* in transgenic WT *A. thaliana* (*Columbia*), *soc1* mutant (SALK 138131C, *Columbia*) and rice (*O. sativa* L. *subsp. japonica* cv. zhonghua 11) plants were analyzed by RT-PCR and confirmed using qRT-PCR. Homozygous transgenic lines of the T3 generation were used for further analyses. For phenotypic observation, transgenic *A. thaliana* seedlings were grown in a greenhouse with 16-h light/8-h dark at 21–23 °C, and transgenic rice seedlings were planted in a growth room owith14-h light/10-h dark at 25–30 °C. All the samples used for gene expression detection were collected in the morning (~5–6 h after the light was turned on) based on a previous study by Lee et al.^43^.

## Supplementary information

supporting information file

Table S1

Table S2

Table S3
