# Supplementary material for: The SOC1-like gene BoMADS50 is associated with the flowering of Bambusa oldhamii
Source: Hortic Res. 2021 Jun 1;8:133. doi: 10.1038/s41438-021-00557-4 (PMC8166863; doi:10.1038/s41438-021-00557-4)
Supplement: Supplementary file 1 — supporting information file [file 41438_2021_557_MOESM1_ESM.docx]

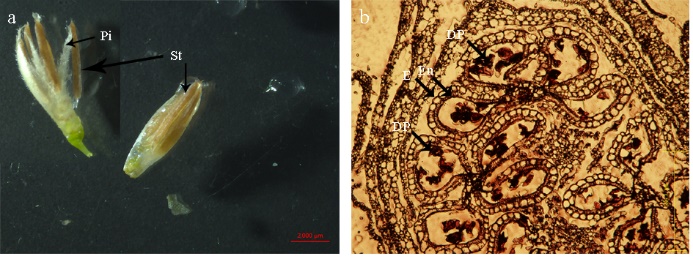


**Figure S1.** The morphology of *Ba. oldhamii* mature floral (a) and its paraffin sections with the scale 25.1 um (b) St: stamen, Pi: pistil, E: epidermis, En: endothecium, DP degraded pollens


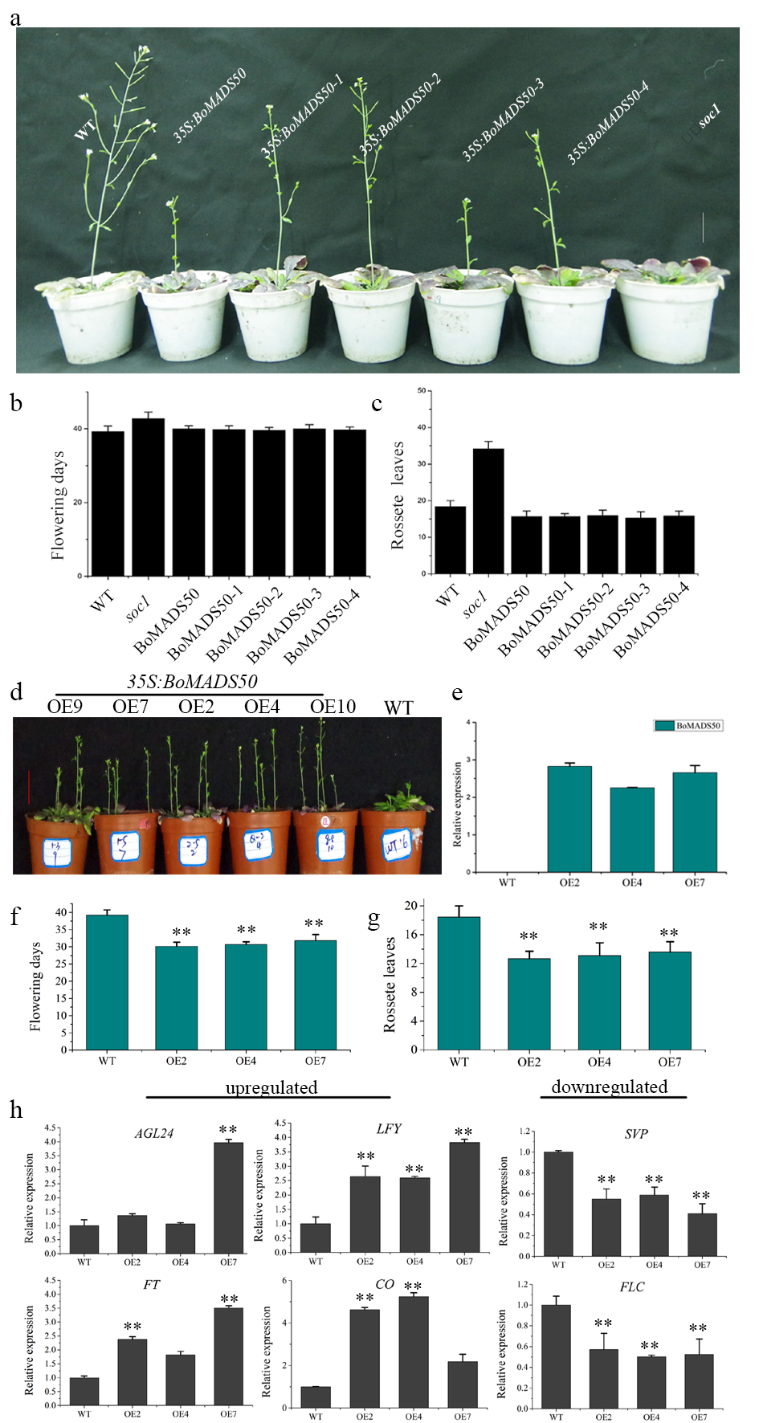


**Figure S2**. Overexpression of *BoMADS50* and *BoMADS50-1/2/3/4* rescued the late-flowering phenotype of *soc1* mutant and caused early flowering in WT Arabidopsis. (a) Morphology of *Arabidopsis* plants overexpressing *BoMADS50* and *BoMADS50-1/2/3/4* at flowering. Bar=2 cm. (b) The flowering days and (c) rosette leaves of WT, *soc1* mutant and three *BoMADS50* and *BoMADS50-1/2/3/4* overexpressed lines. All data are means ± sd (n≥10 independent plants for each line). Asterisks indicate significantly different values (***P* < 0.01). (d) Morphology of *Arabidopsis* plants overexpressing *BoMADS50* at flowering. Bar=2 cm. (e) The expression levels of *BoMADS50* in three independent lines (f) The flowering days and (g) rosette leaves of three *BoMADS50* overexpression lines. All data are means ± sd (n≥10 independent plants for each line). Asterisks indicate significantly different values (**P < 0.01). (h) Analysis of mRNA abundance of the flowering related genes in transgenic *Arabidopsis* lines and WT. Asterisks indicate that the value is significantly different from that of the WT at the same time point (**P*<0.05, ***P* < 0.01).


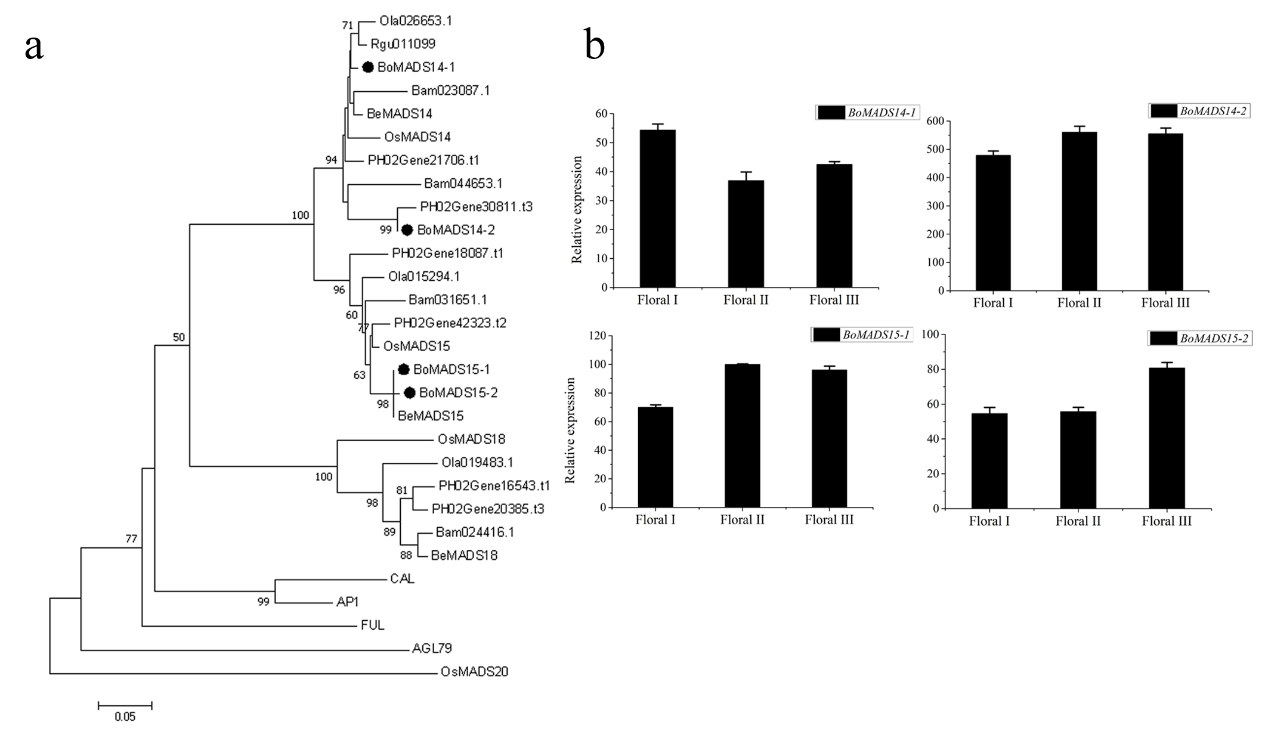


**Figure S3**. Sequence analysis of AP1/FUL proteins in *Ba. oldhamii* (a) A phylogenetic tree of BoMADS14-1, BoMADS14-2, BoMADS15-1 and BoMADS15-2 and AP1/FUL -like proteins from other species was constructed with MEGA 7 using the maximum likelihood (ML) method with 1,000 bootstrap replicates based on a multiple sequence alignment result. Bootstrap values higher than 50% are shown on the nodes. 25 AP1-like proteins were used, four from *A. thaliana* (CAL, AP1, FUL, AGL79), four from rice (OsMADS14, OsMADS15, OsMADS18 and OsMADS20), three from *Ba. edulis* (BeMADS14, BeMADS15, BeMADS18), six from moso bamboo (PH02Gene21706.t1, PH02Gene16543.t1, PH02Gene20385.t3, PH02Gene42323.t2, PH02Gene30811.t3, PH02Gene18087.t1), one from *R. guianensis* (Rgu011099), three from *Ol. latifolia* (Ola026653.1, Ola015294.1 and Ola019483.1) and four from *Bo. amplexicaulis* (Bam023087.1, Bam044653.1, Bam024416.1, Bam031651.1). The BoMADS14-1, BoMADS14-2, BoMADS15-1 and BoMADS15-2 were marked by black circles. (b) The expression patterns of *BoMADS14-1, BoMADS14-2, BoMADS15-1* and *BoMADS15-2* during *Ba. oldhamii* flowering.


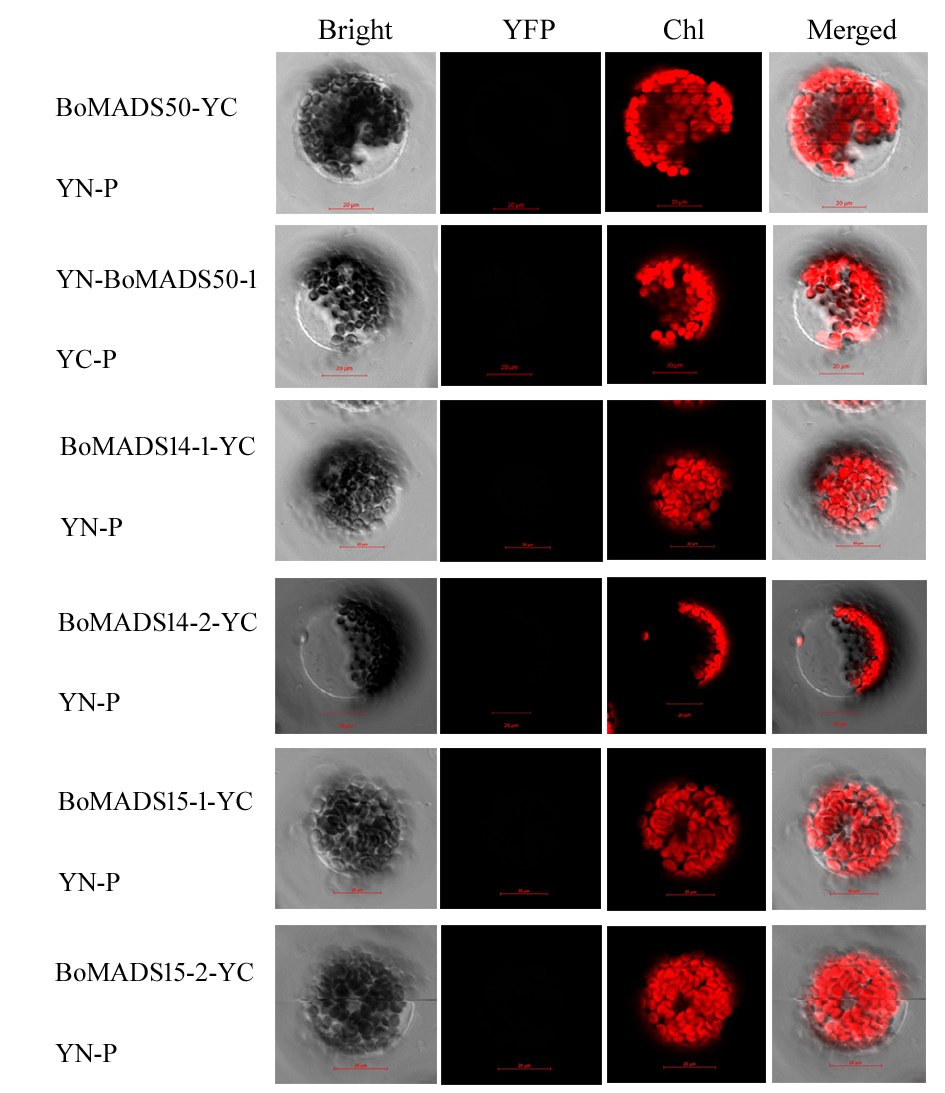


**Figure S4**. The co-transformation of BoMADS50, BoMADS50-1, BoMADS14-1, BoMADS14-2, BoMADS15-1 and BoMADS15-2 with YN-P or YC-P vectors in BiFC assay. No YFP signals could be detected. The bar=20 μm. Bright: bright field; YFP: YFP fluorescence; Chl: chlorophyll autofluorescence; Merged: YFP/bright/Chl field overlay.


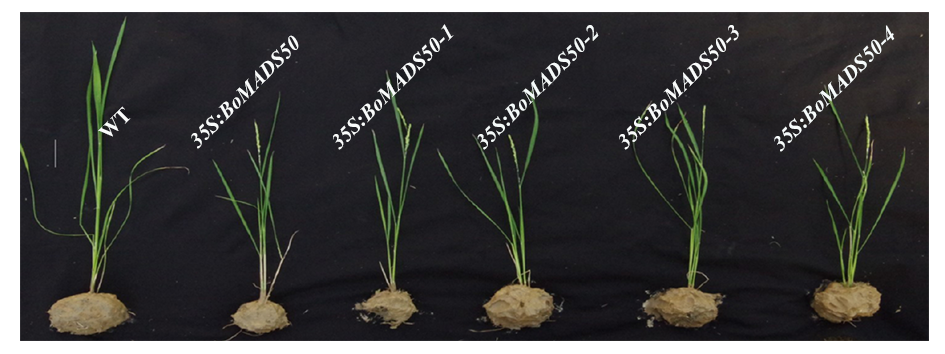


**Figure S5**. Morphology of wild-type and *BoMADS50-1/2/3/4* transgenic rice. Bar=10cm.


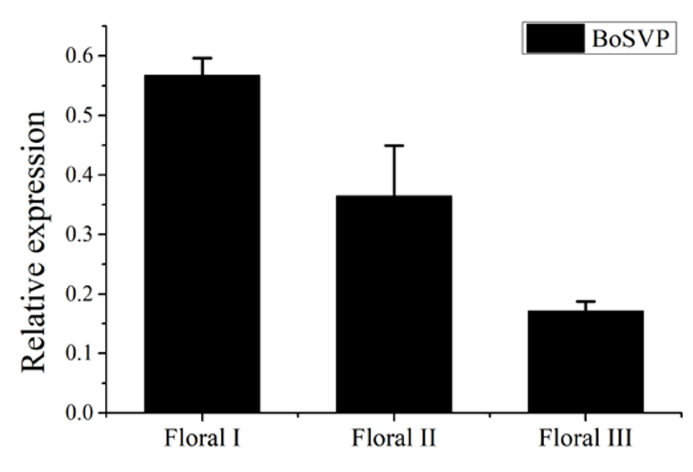


**Figure S6**. The expression pattern of *BoSVP* during *Ba. oldhamii* flowering.
