# Supplementary material for: The SOC1-like gene BoMADS50 is associated with the flowering of Bambusa oldhamii
Source: Hortic Res. 2021 Jun 1;8:133. doi: 10.1038/s41438-021-00557-4 (PMC8166863; doi:10.1038/s41438-021-00557-4)
Supplement: Supplementary file 2 — Table S1 [file 41438_2021_557_MOESM2_ESM.pdf]

Primer name	Sequences(5'-3')	
**For *BoMADS50* gene clone**		
S-F	GGGAAGACGCAGATGAAGCGGAT	conserved fragment
S-R	ACTTGCTCTATATCTTGCTGTAC	clone
AP	GGCCACGCGTCGACTAGTACTTTTTTTTTTTTTTTTT	3'-RACE
AUAP	GGCCACGCGTCGACTAGTACTGSP	
3'GSP1	GCACCAAGACAGTAGAGCAAGAT	
3'GSP2	GATGCTGAGGGCTTGGCAA	
AP	GCTTATTCTGGGTATGCTT	5'-RACE
APP	GGCCACGCGTCGACTAGTACGGGIIGGGIIGGGIIG	
AUAP	GGCCACGCGTCGACTAGTAC	
5'GSP1	GAGAAGACGACGAGGGCGA	
5'GSP2	CCGCTTCATCTGCGTCTTGC	
QGSP-F	GTGGTCTGGATTTGGGGGCT	For full length clone
QGSP-R	CGGTTGATTTTAGGGTGTCATAGC	
BoMADS50-F	ATGGTGCGGGGGAAGACGCAGAT	For ORF clone
BoMADS50-R1	TTATCCTGACTTATCTGTTGTCG	
		For nsSNP detection
BoMADS50-R2	TCAAGCATGGTTATCCTGAC	on DNA
DNA-PCR-F1	GTTTCTGTCTGCTGTTTGTTGCATG	
DNA-PCR-R1	GAAAAAAAAATAAAAACCTCATGAGTTGCA	
DNA-PCR-F2	GGAACGAATAAAAAACCAGCCAAATCAGCG	
DNA-PCR-R2	TTTTGGTGAGTCCCCCCTTGCCTTC	
**For *AP1/FUL* gene clone**		
BoMADS14-1F	ATGGGGCGCGGGAAGGTGCA	
BoMADS14-1R	TCATCCGTTAATGTGGCTCACCATCCATG	
BoMADS14-2F	ATGGGGCGCGGGAAGGTGCA	
BoMADS14-2R	TCATGAAGGACGAGGAAGAGTCTGGT	
BoMADS15-1F	ATGGGTCGCGGCAAGGTG	
BoMADS15-1R	TTAAGCATTGTGGTGGCTCAGCATCCATG	
BoMADS15-2F	ATGGGTCGCGGCAAGGTG	
BoMADS15-2R	TTAAGCATTGTGGTGGCTCAGCATC	
**For overexpression/subcellular localization/dual-luciferase reporter assay**		
1300-BoMADS50/-1-F	gagctcggtacccggggatccATGGTGCGGGGGAAGACG	
1300-BoMADS50-R	catgtcgactctagaggatccAGCATGGTTATCCTGACTATCTG	
1300-BoMADS50-1-R	catgtcgactctagaggatccTCCTGACTTATCTGTTGTCGCC	
**For dual-luciferase reporter assay**		
1300-BoMADS14-1F:	gagctcggtacccggggatccATGGGGCGCGGGAAGGTGCA	
1300-BoMADS14-1R:	catgtcgactctagaggatccTCCGTTAATGTGGCTCACCATCCATG	
pGreenII-ProBoMADS50F	ctcgaggtcgacggtatcgataCCAACAGTAACACCACAAACACAGT	
pGreenII-ProBoMADS50R	ccgctctagaactagtggatcCTTGGACCAACCGACGGAT	
pGreenII-ProBoSVPF	ctcgaggtcgacggtatcgataGGAGTACGTACTCCTACCTCTCATA	
pGreenII-ProBoSVPR	ccgctctagaactagtggatcAACCCTAACTGGACGGCATGG	

**For Y2H**	
BD-BoMADS50/-1-F	atggaggccgaattcccggggATGGTGCGGGGGAAGACG
BD-BoMADS50-R	ccgctgcaggtcgacggatccTCAAGCATGGTTATCCTGACTATCTG
BD-BoMADS50-1-R	ccgctgcaggtcgacggatccTTATCCTGACTTATCTGTTGTCGCC
**For Y1H&Y2H**	
AD-BoMADS50/-1-F	gagtggccattatggcccgggATGGTGCGGGGGAAGACG
AD-BoMADS50-R	gccgacatgtttttcccgggTCAAGCATGGTTATCCTGACTATCTGv
AD-BoMADS50-1-R	gccgacatgtttttcccgggTTATCCTGACTTATCTGTTGTCGCC
AD-BoMADS14-1F	gagtggccattatggcccgggATGGGGCGCGGGAAGGTGCA
AD-BoMADS14-1R	gccgacatgtttttcccgggTCATCCGTTAATGTGGCTCACCATCCATG
AD-BoMADS14-2F	gagtggccattatggcccgggATGGGGCGCGGGAAGGTGCA
AD-BoMADS14-2R	gccgacatgtttttcccgggTCATGAAGGACGAGGAAGAGTCTGGT
AD-BoMADS15-1F	gagtggccattatggcccgggATGGGTCGCGGCAAGGTG
AD-BoMADS15-1R	gccgacatgtttttcccgggTTAAGCATTGTGGTGGCTCAGCATCCATG
AD-BoMADS15-2F	gagtggccattatggcccgggATGGGTCGCGGCAAGGTG
AD-BoMADS15-2R	gccgacatgtttttcccgggTTAAGCATTGTGGTGGCTCAGCATC
**For Y1H**	
His-ProBoMADS50frag-F	GAATTCATGCGCCGCCTGCCAGT
His-ProBoMADS50frag-R	ACGCGTCTTGGACCAACCGACGGAT
His-ProBoSVPfrag-F	GAATTCCGTCCTATATATATATTGTTTATC
His-ProBoSVPfrag-R	ACGCGTACTCGCCCGACGCCATTGGT
**For BiFC**	
BD-BoMADS50/-1-F	agatctcgagctcaagcttcgATGGTGCGGGGGAAGACG
BD-BoMADS50-R	ggtaccgtcgactgcagaattTCAAGCATGGTTATCCTGACTATCTGv
BD-BoMADS50-1-R	ggtaccgtcgactgcagaattTTATCCTGACTTATCTGTTGTCGCC
BiFC-BoMADS14-1F	agatctcgagctcaagcttcgATGGGGCGCGGGAAGGTGCA
BiFC-BoMADS14-1R	ggtaccgtcgactgcagaattTCATCCGTTAATGTGGCTCACCATCCATG
BiFC-BoMADS14-2F	agatctcgagctcaagcttcgATGGGGCGCGGGAAGGTGCA
BiFC-BoMADS14-2R	ggtaccgtcgactgcagaattTCATGAAGGACGAGGAAGAGTCTGGT
BiFC-BoMADS15-1F	agatctcgagctcaagcttcgATGGGTCGCGGCAAGGTG
BiFC-BoMADS15-1R	ggtaccgtcgactgcagaattTTAAGCATTGTGGTGGCTCAGCATCCATG
BiFC-BoMADS15-2F	agatctcgagctcaagcttcgATGGGTCGCGGCAAGGTG
BiFC-BoMADS15-2R	ggtaccgtcgactgcagaattTTAAGCATTGTGGTGGCTCAGCATC
**For RT-qPCR**	
BoMADS50-F	GGAGATGACGTTGCTCAAG
BoMADS50-R	TGTCAGAGCGATGTCTGCC
BoMADS14-1F	CTGAAGGCGAAGGTTGAGAC
BoMADS14-1R	TGCTTCTCCACGAGTTCCTT
BoMADS14-2F	CTGAAGGCGAAGGTTGAGAC
BoMADS14-2R	GCTGGCTCTTTCTGGATCTG

BoMADS15-1F	GGAGAGGCAGAAGGTGTTCA
BoMADS15-1R	TAAGCATTGTGGTGGCTCAG
BoMADS15-2F	TCATGGGAGAGGATCTGGAG
BoMADS15-2R	CATAAGGCGGCTCTTTCTTG
BoSVPF	TCGGCCTCATCGTTTTCTCC
BoSVPR	GTTCAGTCTGTGCAGCTCCT
NTB-F	TCTTGTTTGACACCGAAGAGGAG
NTB-R	AATAGCTGTCCCTGGAGGAGTTT
FLC-F	AGCCAAGAAGACCGAACTCA
FLC-R	TTTGTCCAGCAGGTGACATC
SVP-F	CAAGGACTTGACATTGAAGAGCTTCA
SVP-R	CTGATCTCACTCATAATCTTGTCAC
FT-F	AGACGTTCTTGATCCGTTTA
FT-R	GTAGATCTCAGCAAACTCGC
CO-F	CCGGGTCTGCGAGTCATG
CO-R	GGCATCATCTGCCTCACACA
AGL24-F	GAGGCTTTGGAGACAGAGTCGGTGA
AGL24-R	AGATGGAAGCCCAAGCTTCAGGGAA
LFY-F	ATCGCTTGTCGTCATGGCTG
LFY-R	GCAACCGCATTGTTCCGCTC
Atactin1-F	CAGACACGGCGATGGCGATA
Atactin1-R	GCTTTCTCTCAAGGGTTTCTGGGT
RFT1-F	TTCCTGGTACCACTGGAGCA
RFT1-R	GTCTCAGCTTAGCTATAGCT
OsMADS50-F	AAAGCTGACGCTGATGGTTTG
OsMADS50-R	GTTTCGACATCCATGTTGTC
OsMADS56-F	GACCGCTATAAAGCATACACA
OsMADS56-R	TCATGTGGTTAGCCACCAGC
Ehd2-F	AACAGCAGCAGCATCACTAC
Ehd2-R	AGCAGGAGTGGTGGAGAATG
Ehd1-F	CGACAAAACACAAGACCACCCT
Ehd1-R	CCTGTTTGTCTGAATCCCATCG
Ghd7-F	GCTTGAACCCAAACACGG
Ghd7-R	CTCATCTCGGCATAGGCTT
Oshd1-F	GGTTATGGAGTTGTGGGAGCAGAC
Oshd1-R	AGTGAAGGGACATCTGAAGCGAGG
OsUbq-F	ACCACTTCGACCGCCACTACT
OsUbq-R	ACGCCTAAGCCTGCTGGTT
