# Supplementary material for: The SOC1-like gene BoMADS50 is associated with the flowering of Bambusa oldhamii
Source: Hortic Res. 2021 Jun 1;8:133. doi: 10.1038/s41438-021-00557-4 (PMC8166863; doi:10.1038/s41438-021-00557-4)
Supplement: Supplementary file 3 — Table S2 [file 41438_2021_557_MOESM3_ESM.pdf]

Table S2. Flowering days of wild-type and *BoMADS50-1/2/3/4* transgenic rice

WT	35S::BoMADS50-1	35S::BoMADS50-2	35S::BoMADS50-3	35S::BoMADS50-4
76.62±2.02	58.87±9.08	61.08±11.00	52.23±0.53	71.06±0.18
