# Supplementary material for: The SOC1-like gene BoMADS50 is associated with the flowering of Bambusa oldhamii
Source: Hortic Res. 2021 Jun 1;8:133. doi: 10.1038/s41438-021-00557-4 (PMC8166863; doi:10.1038/s41438-021-00557-4)
Supplement: Supplementary file 4 — Table S3 [file 41438_2021_557_MOESM4_ESM.pdf]

Table S3. Analysis of transposable elements in ORF region of *BoMADS50*.

Gene name	Transposable element	From	To	Class	Similarity
BoMADS50	REP-9_OS	1	188	Interspersed_Repeat	0.8667
